# *Plasmodium* dihydrofolate reductase is a second enzyme target for the antimalarial action of triclosan

**DOI:** 10.1038/s41598-018-19549-x

**Published:** 2018-01-18

**Authors:** Elizabeth Bilsland, Liisa van Vliet, Kevin Williams, Jack Feltham, Marta P. Carrasco, Wesley L. Fotoran, Eliana F. G. Cubillos, Gerhard Wunderlich, Morten Grøtli, Florian Hollfelder, Victoria Jackson, Ross D. King, Stephen G. Oliver

**Affiliations:** 1 Cambridge Systems Biology Centre & Department of Biochemistry, University of Cambridge, Cambridge, UK; 2 Department of Structural and Functional Biology, Institute of Biology, UNICAMP, Campinas, SP Brazil; 3 Department of Computer Science, Aberystwyth University, Aberystwyth, UK; 4 Department of Chemistry & Molecular Biology, University of Gothenburg, Gothenburg, Sweden; 5 Department of Parasitology, Institute of Biomedical Sciences, University of São Paulo, São Paulo, SP Brazil; 6 Manchester Institute of Biotechnology, University of Manchester, Manchester, UK

## Abstract

Malaria, caused by parasites of the genus *Plasmodium*, leads to over half a million deaths per year, 90% of which are caused by *Plasmodium falciparum*. *P*. *vivax* usually causes milder forms of malaria; however, *P*. *vivax* can remain dormant in the livers of infected patients for weeks or years before re-emerging in a new bout of the disease. The only drugs available that target all stages of the parasite can lead to severe side effects in patients with glucose-6-phosphate dehydrogenase (G6PD) deficiency; hence, there is an urgent need to develop new drugs active against blood and liver stages of the parasite. Different groups have demonstrated that triclosan, a common antibacterial agent, targets the *Plasmodium* liver enzyme enoyl reductase. Here, we provide 4 independent lines of evidence demonstrating that triclosan specifically targets both wild-type and pyrimethamine-resistant *P*. *falciparum* and *P*. *vivax* dihydrofolate reductases, classic targets for the blood stage of the parasite. This makes triclosan an exciting candidate for further development as a dual specificity antimalarial, which could target both liver and blood stages of the parasite.

## Introduction

Over half a million deaths per year result from malaria, caused by parasites of the genus *Plasmodium*. Approximately 90% of these deaths (generally of children in Africa) are caused by *Plasmodium falciparum*, the parasite responsible for the most severe forms of the disease. However, *Plasmodium vivax* is the malaria parasite with the largest global distribution, threatening 2.5 billion people - mainly in South America and Asia^1^. Although *P*. *vivax* usually causes milder forms of malaria, it is still a killer^2^. Moreover, *P*. *vivax* can remain dormant in the livers of infected patients for weeks or years before re-emerging in a new bout of the disease^3^.

There is an urgent need to develop new anti-malarial drugs as resistance has arisen against all existing drugs^4–6^, and there is no immediate prospect of an effective vaccine^7^. Primaquine is the only drug in use that targets all stages of the parasite. However, it greatly increases the risk of hemolysis in patients with glucose-6-phosphate dehydrogenase (G6PD) deficiency, as does the promising new drug, tafenoquine^1^. G6PD deficiency is prevalent in areas where malaria is endemic since it offers some protection against infection^8^.

Triclosan is a simple antimicrobial agent that has been used for more than 40 years, and which is safe enough to be incorporated into consumer products from toothpastes to toys^9,10^. In 2001, it was reported that triclosan inhibits the *in vitro* propagation of *P*. *falciparum*^11^. Since then, triclosan and its analogs have been repeatedly shown to inhibit the growth of blood-stage *P*. *falciparum* in culture^12–19^. It was assumed that the mechanism of action of triclosan against *Plasmodium* was the same as that against bacteria - inhibition of enoyl reductase in the FAS-II^11^ fatty-acid synthesis pathway. Indeed, phenotypical, biochemical, structural evidence, and *in silico* simulations demonstrated that triclosan is a strong inhibitor of the *P*. *falciparum* enoyl reductase (*Pf*ENR)^13,20,21^. However Yu *et al*.^20^ showed convincingly that the FAS-II pathway is not important for the propagation of *P*. *falciparum* in erythrocytes and so inhibition of *Pf*ENR cannot explain the effect of triclosan on blood-stage parasites.

Efforts to optimize the compound for use as an antimalarial showed no correlation between *Pf*ENR inhibition *in vitro* and triclosan’s activity against the living parasite^21,22^. Since it has been demonstrated that *Pf*ENR is important for the liver stage of the parasite, but not for the erythrocyte stage^22,23^, action against a different target must be responsible for triclosan’s inhibition of the growth of blood-stage *P*. *falciparum*.

We have developed an automated yeast-based assay for use in high-throughput screens for compounds that are selectively active against target enzymes from parasites^24,25^. Our assay is based on replacing yeast genes essential for growth, with coding sequences specifying orthologous proteins from either parasites or humans, making the strains dependent on the activity of the parasite or the human enzyme in order to grow^25^. These strains are then labeled with different fluorescent proteins and pooled to allow monitoring of the growth, in real time, of each strain in competition with the others.

We carried out growth competition experiments between 3 yeast strains dependent on a DHFR enzyme from *P*. *falciparum*, *P*. *vivax*, or the human DHFR (these three strains were differentiated from one another by each expressing either the fluorescent proteins mCherry, Venus, or Sapphire^26,27^). These competitions took place in single wells of microtiter trays, enabling a Robot Scientist to screen thousands of different compounds and identify candidates with no general cytotoxicity, but which inhibited the parasite target without affecting its human counterpart^24,28^.

## Results and Discussion

**Figure 1:**
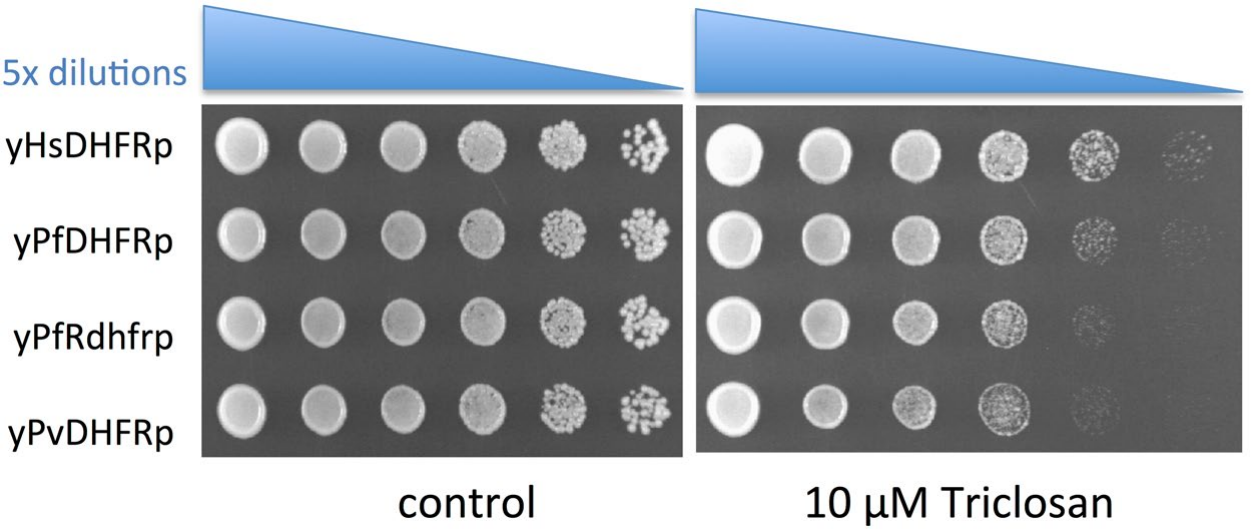
Triclosan specifically inhibits *Plasmodium* DHFRs in yeast. Yeast strains expressing either *Homo sapiens* DHFR (yHsDHFR), *Plasmodium falciparum* wild-type DHFR (yPfDHFR), pyrimethamine-resistant *P*. *falciparum* DHFR (yPfRdhfr), or *P*. *vivax* DHFR were cultivated overnight and dilutions 5, 25, 125, 625 and spotted onto YNB-glucose-agar plates containing 0 or 10 μM irgasan (triclosan).

Dihydrofolate reductase (DHFR) is an enzyme that catalyzes the NADPH-dependent reduction of dihydrofolate to tetrahydrofolate. This reaction is essential for the *de novo* synthesis of purines and certain amino acids, making DHFR essential for rapid growth, and is the target for the action of the important antimalarial drugs pyrimethamine and proguanil. Employing our yeast-based high-throughput screening approach^24^, we screened the Johns Hopkins Library of FDA-approved compounds against yeast strains expressing human DHFR (*Hs*DHFR), *P*. *falciparum* DHFR (*Pf*DHFR), *P*. *vivax* DHFR (*Pv*DHFR) as well as the pyrimethamine-resistant *P*. *vivax* and *P*. *falciparum* DHFRs (*Pv*Rdhfr and *Pf*Rdhfr)^28^. The automated screen identified triclosan (Eve ID 21658, JHU-10450) as a specific inhibitor of *P*. *vivax* and *P*. *falciparum* DHFRs, including the pyrimethamine-resistant variants of the two enzymes. The Robot Scientist Eve prepared titration experiments with 1 to 20 μM of triclosan and successfully confirmed the hits. We prepared serial dilutions of yeast cultures expressing the human or *Plasmodium* DHFRs and spotted those onto agar plates containing 10 μM of triclosan, further confirming the specificity of the drug for the parasite target (Fig. 1).

**Figure 2:**
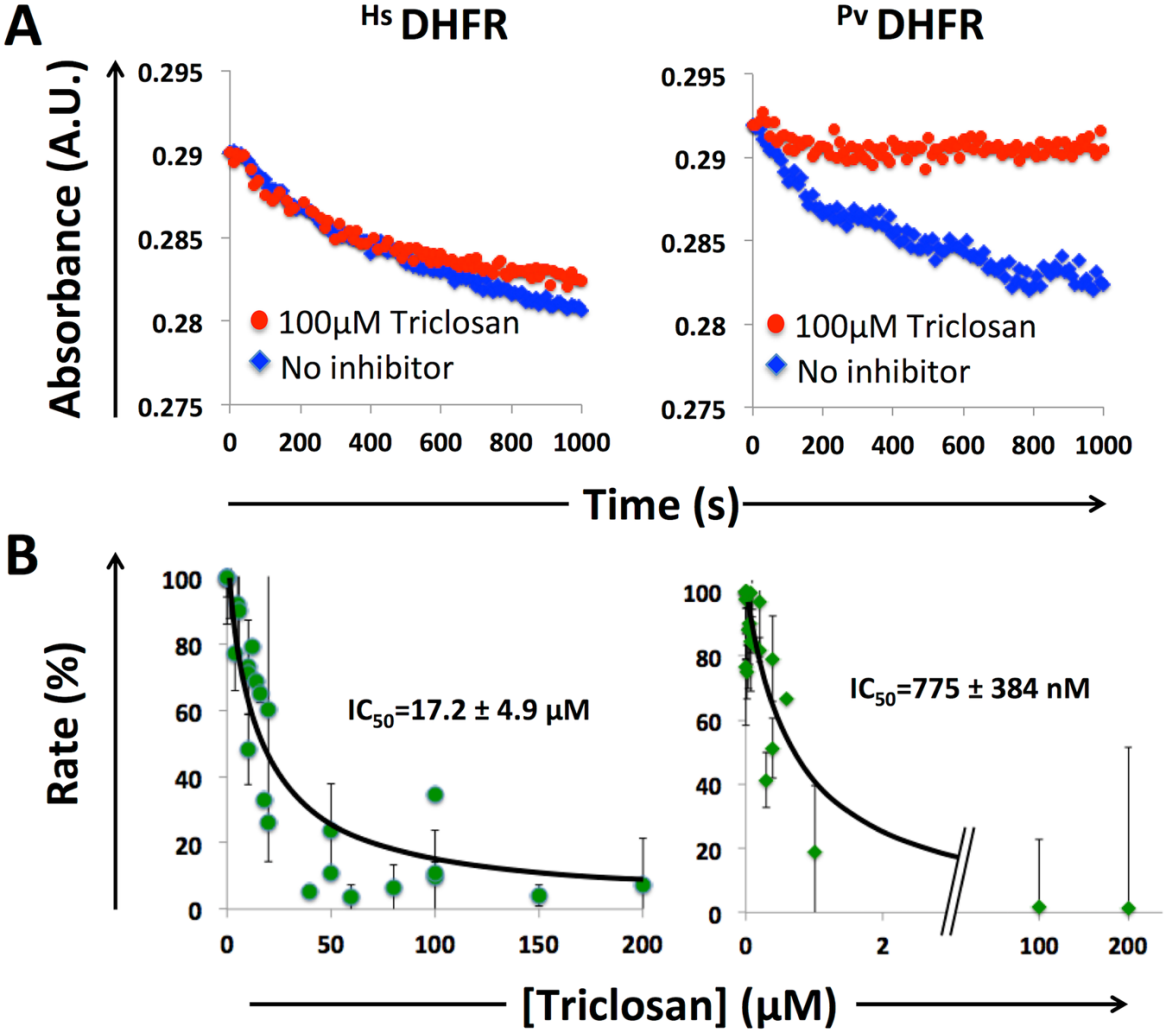
Triclosan inhibits *Plasmodium* DHFR *in vitro*. (**A**) DHFR catalyzes the reduction of dihydrofolate (DHF) to tetrahydrofolate (THF), coupled with the oxidation of nicotinamide adenine dinucleotide phosphate (NADPH). This reaction was followed for *Hs*DHFR and *Pv*DHFR in the absence (blue) or presence (red) of 100 µM Triclosan at 340 nm. (**B**) Initial rates of reaction were determined and used to derive IC_50_ values, with the standard deviation calculated from multiple kinetic runs (n = 3). To deal with variations in enzyme activity in different preparations, kinetic data were normalized as relative activities and fitted to % activity = % activity_max_/(1 + [triclosan]/IC_50_). Reaction conditions: [triclosan] = 0–200 μM, [*Hs*DHFR] = 30.5 nM, [*Pv*DHFR] = 18.5 nM, [Tris/HCl] = 0.1 M, pH 7.4, [DHF] = [NADPH] = 100 μM, 21 °C.

We expressed the human and *P*. *vivax* DHFRs in *E*. *coli*, and purified the proteins using methotrexate affinity columns. We then performed *in vitro* enzyme assays and observed that the *P*. *vivax* DHFR is >20× more sensitive to triclosan (IC_50_ of 775 ± 384 nM) than the human enzyme (IC_50_ of 17.2 ± 4.9 μM) (Fig. 2).

To further validate DHFR as a target of triclosan in *Plasmodium*, we transfected blood-stage *P*. *falciparum* NF54 with a plasmid expressing the human DHFR enzyme (*Hs*DHFR). If the blood-stage target of triclosan is indeed the *Plasmodium* DHFR, expression of *Hs*DHFR (which our yeast and *in vitro* assays showed to be resistant to triclosan) should protect the parasite against the compound.

**Figure 3:**
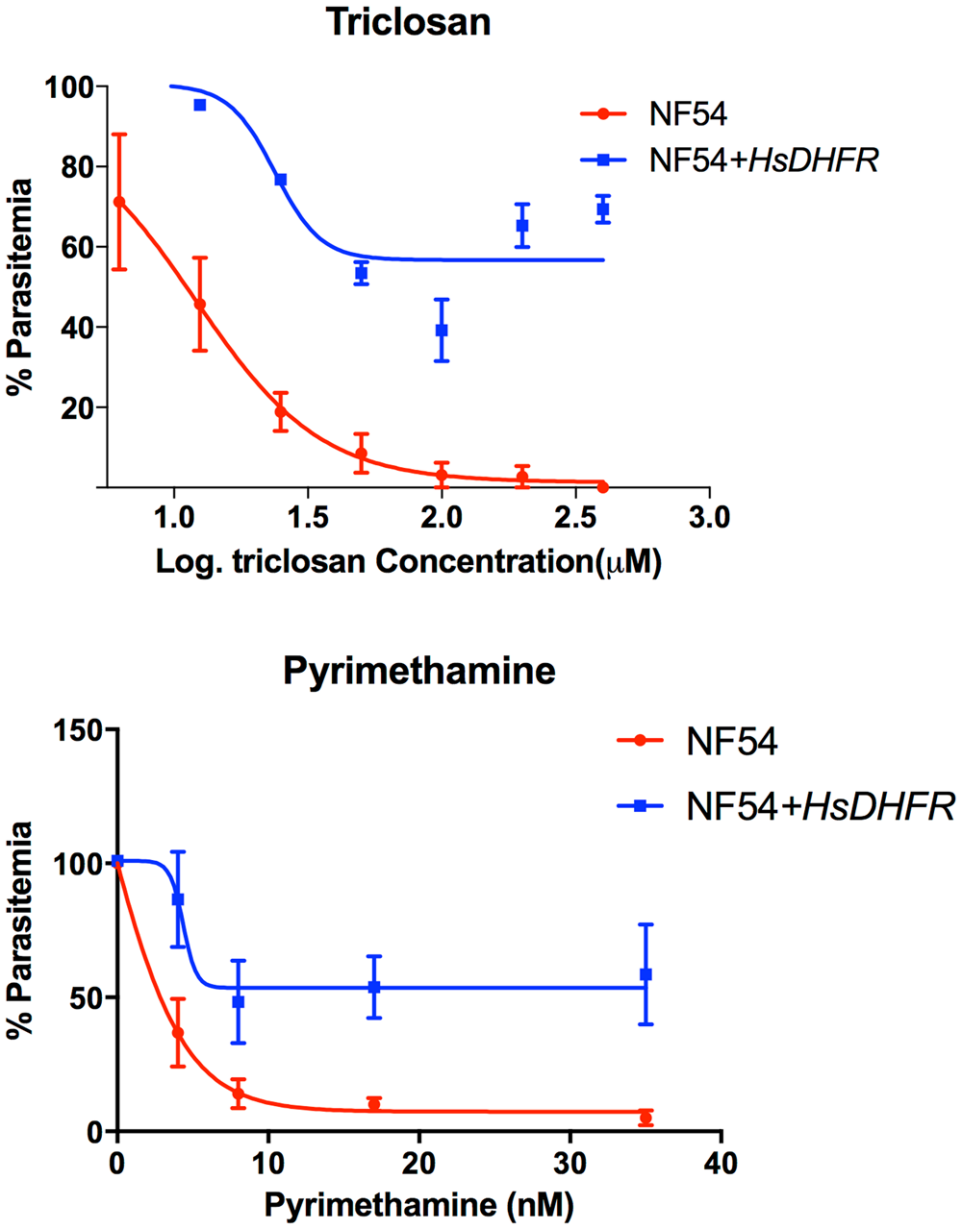
Human DHFR protects *Plasmodium* from triclosan. *P*. *falciparum* NF54 strain was transfected with a plasmid expressing human dihydrofolate reductase, and treated with either triclosan or pyrimethamine for 48 h. Relative parasitemias were calculated following JC1 staining and flow cytometry analysis. Error bars indicate SEM.

We found that our transfected *Plasmodium* strain was more tolerant to exposure to triclosan than the wild type (Fig. 3), consistent with DHFR being the compound’s target when the parasite is within the erythrocyte. The difference in the rate of progression of the parasitemia in wild-type and transfected strains was compared at a range of triclosan concentrations and found to be statistically significant (SEM). It should be noted that, in contrast to the *in vitro* enzyme inhibition kinetics, those for growth inhibition are complex. This is likely to be due to the fact that our parasite lines have been transfected with episomal constructs bearing the coding sequence for human DHFR. The resulting range of plasmid copy numbers would result in differences in the levels of human DHFR between individual *Plasmodium* cells in the population^29^. The consequence is that a considerable proportion of cells do not express human DHFR at levels that are sufficiently high to confer drug resistance.

**Figure 4:**
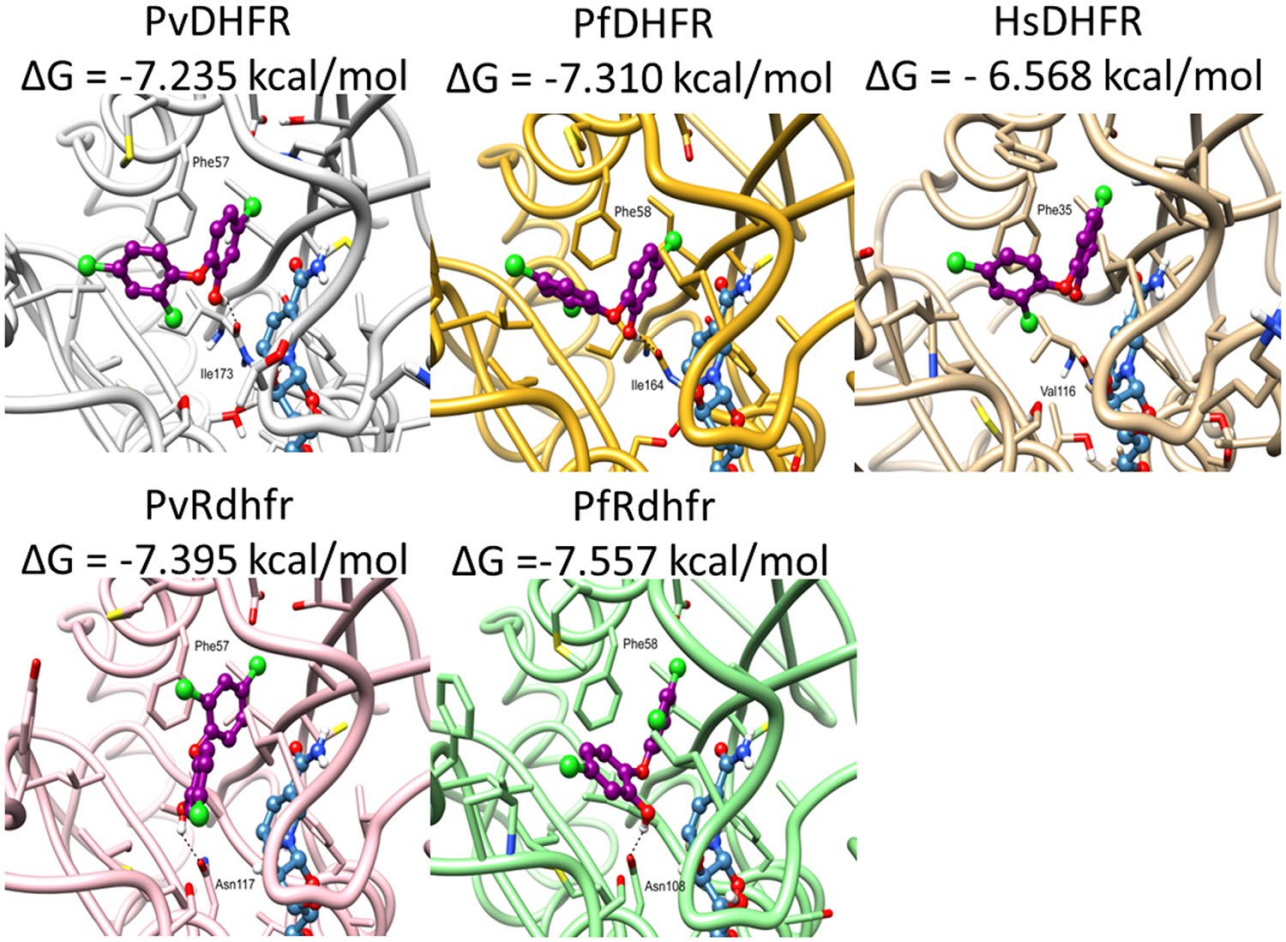
Differential binding of triclosan to *Plasmodium* and human DHFRs. Predicted binding poses of triclosan (in purple) bound to the active site of DHFRs. Wild-type *P*.*vivax* DHFR (PDB ID: 2BL9), double mutant *P*. *vivax* DHFR (PDB ID: 2BLC), *P*. *falciparum* DHFR (PDB ID: 4DPD), quadruple mutant *P*. *falciparum* DHFR (PDB ID: 4DP3), and human DHFR (PDB ID: 3NTZ). NADPH is colored in blue; hydrogen bonds are represented by black dashed lines.

Finally, triclosan was docked into the X-ray structures of human and *Plasmodium* DHFRs (Fig. 4). Although triclosan displayed slightly altered binding poses in the different parasitic DHFRs, the inhibitor showed the same interactions with all four DHFRs, indicating comparable affinity (binding free energies in the range −7.24 and −7.56 kcal/mol). In contrast, triclosan was not interacting with the *Hs*DHFR through hydrogen bonding, resulting in reduced affinity (∆*G* = −6.57 kcal/mol) and supporting the contention that triclosan discriminates between the *Plasmodium* and human enzymes.

DHFR inhibitors are routinely used as prophylactic drugs and are given to over a million children during the malaria season. However, DHFR inhibitors are no longer a standard treatment for the disease because of the evolution of drug-resistant variants of *Plasmodium*. Extensive efforts to discover a DHFR-targeted antimalarial that is effective against pyrimethamine-resistant strains have yet to produce a clinically approved entity, thus a novel Triclosan-derived compound with activity against drug-resistant forms of DHFR is of high potential value.

The presence of triclosan in consumer products in concentrations 2500-fold above the present IC_50_^30^, suggests that it can be administered in quantity and short-term toxicity effects can be disregarded, notwithstanding possible long-term hazards^31^. This tolerance is important, as delivering effective concentrations *in vivo* may be challenging since data on the effect of administering triclosan to mice infected with *P*.*berghei* are contradictory^11,18^, with sub-cutaneous delivery being more effective than oral administration^18^. It may be that further engineering of triclosan by synthetic chemistry is necessary to improve delivery and half-life, but with regard to affinity triclosan is a viable non-toxic lead with a mechanism based on hitherto undiscovered selectivity. A number of triclosan analogs have already been synthesized^16,21^, demonstrating the ample opportunity for its improvement for use as an antimalarial.

In this work, we have provided four different lines of evidence (screens using recombinant yeast strains, enzyme assays, growth assays with blood-stage *Plasmodium*, and *in silico* simulations of drug-enzyme interactions) demonstrating that triclosan specifically targets the DHFR enzyme of both *P*. *vivax* and *P*. *falciparum*, and that it is capable of selectively inhibiting both the wild-type and pyrimethamine-resistant enzymes compared to human DHFR. Our conclusion that triclosan targets *Plasmodium* DHFR also explains much of the inconsistency in the literature regarding the efficacy of triclosan^22^: *Hs*DHFR is commonly used as a *Plasmodium* selective marker as it provides pyrimethamine resistance, and controls for this confounding factor are not always performed.

Given the earlier evidence that triclosan inhibits ENR, it may be that triclosan is an example of “polypharmacology”^32^, poised for multitarget therapies. The totality of data on this compound suggests that it is capable of inhibiting blood-stage *Plasmodium*, via its action against DHFR, and the liver stage of the parasite, via the inhibition of a key enzyme in fatty-acid synthesis^20^. Interestingly, polyphenol compounds such as epigallocathechin gallate (EGCG), also inhibit fatty acid synthesis and have antiplasmodial activity^33^, and have demonstrated antifolate activity^34^. Given the recent success in developing drug candidates with multi-stage activity^35^, it is realistic to envision the development of a new dual-action drug, based on triclosan, that is effective against both the blood and liver stages of wild-type and pyrimethamine-resistant *Plasmodium* species. The fact that triclosan has two distinct enzyme targets in different domains of metabolism may militate against the development of resistance. All of this engenders the hope that our discovery of a second enzyme target for triclosan may prove to be the first step in the development of a novel class of antimalarials.

## Methods

### Yeast strains and fluorescent plasmids

All yeast strains and marker plasmids used in this work are described in Bilsland *et al*.^24,25^. Briefly: plasmids, bearing genes for the expression of fluorescent proteins, were constructed by replacing the coding region of yEmRFP from yEpGAP-Cherry with Venus or Sapphire, and replacing the *URA3* marker with *LEU2*. These three fluorescent proteins have very distinct excitation and emission wavelengths: mCherry (λ_ex_ 580 nm; λ_em_ 612 nm), Sapphire (λ_ex_ 405 nm; λ_em_ 510 nm) and Venus (λ_ex_ 500 nm; λ_em_ 540 nm).

A strain expressing the drug-resistant *P*. *vivax* DHFR (yPvRdhfr) was constructed by making the following site-directed mutations in the coding sequence for the *P*. *vivax* enzyme: S58R, S117N and I173L. This plasmid was transformed into *dfr1Δ/DFR1* a yeast strain with the BY4743 genetic background. The strain was sporulated and *MATα* haploids were selected and used in drug screens. The drug-resistant yPfRdhfr strain, which is a triple mutant for residues N51I, C59R, and S108N, was generated in a similar manner.

### Library-screening assays and hit confirmation using the Robot Scientist Eve

Automated screens were performed as described in Williams *et al*.^28^. Starter cultures of individual strains, labeled with different fluorescent proteins, were grown to stationary phase in YNB-glucose (0.67% yeast nitrogen base without amino acids, 2% ammonium sulphate and 2% glucose) with the appropriate auxotrophic supplements. An aliquot (1 mL) of each pre-culture was inoculated into 100 mL of fresh medium. Pools of three strains were incubated for 4 h at 30 °C, with shaking, to ensure exponential growth. Doxycycline (Sigma-Aldrich) (5 μg/mL) was then added to the culture to reduce expression of the gene for the heterologous enzymes^24^. The culture was attached to a Thermo Combi multidrop within the Robot Scientist Eve work cell^24^. The culture was stirred continuously and maintained at 23 °C during assay plate set-up.

High-throughput drug screens were performed by the Robot Scientist Eve using the mixed cultures described above and the ~1,600 FDA- and foreign- approved drugs from the Johns Hopkins University Clinical Compound Library. Strains were grown in competition in the presence of a library compound, and the relative growth rates used to estimate the activity of the drug against the parasite target. For hit confirmation assays, the Robot Scientist Eve prepared plates with eight replicates of eight different compounds, at six different concentrations (0, 1, 2.5, 5, 10, 20 μM), and 64 negative control wells.

### Recombinant protein expression and purification

Synthetic genes encoding versions of the *Homo sapiens* and *P*. *vivax* dihydrofolate reductases^25^, tagged with maltose-binding protein (MBP) and polyhistidine (HIS) sequences, were cloned into the *Bam*HI/*Nsi*I sites of pMAT10 (Keily Littlefield and Darerca Owen, personal communication). *Escherichia coli* BL21 (DE3) transformants, harboring each of the plasmids, were grown in LB medium and recombinant protein expression was induced with 0.4 mM isopropyl-1-β-D-thio-galactopyranoside (IPTG) for 16 hours at 15 °C. Cultures were centrifuged at 4000 rpm for 10 min, the supernatant discarded, and cell pellets resuspended in 1/50th of the culture volume in 20 mM Tris-HCl, pH 7.4, 300 mM NaCl and Roche Complete EDTA-free Protease Inhibitor tablets, DNase I (RNase-free; Thermo-Scientific 2 U.mL^−1^), 1 mM Tris(2-carboxyethyl)phosphine hydrochloride (Sigma). Cell suspensions were passed three times through an EmulsiFlex-C5 to facilitate cell lysis.

Cell lysates were centrifuged at 20,000 rcf for 50 min at 8 °C. MBP_HIS-tagged proteins were purified in HIS-Select® Nickel Affinity Gel (Sigma-Aldrich) gravity-flow columns. MBP_HIS-tagged DHFRs were dialyzed into Thrombin Cleavage Buffer (20 mM Tris-HCl, pH 8.4, 2.5 mM CaCl_2_ and 150 mM NaCl) and incubated for 5 hours at 20 °C with 24 mM lactose and 5 U/mL bovine thrombin (GE Healthcare). Cleavages were terminated by raising the NaCl concentration of the solution to 0.5 M and binding to 1 mL HiTrap Benzamidine FF columns (GE Healthcare) pre-equilibrated with High Salt Thrombin Buffer (20 mM Tris-HCl, pH 8.4, 2.5 mM CaCl_2_ and 500 mM NaCl). Cleaved samples were dialyzed into Methotrexate Equilibration Buffer (50 mM Tris- HCl, pH 8.0, 2 mM EDTA, 0.5 M NaCl, 2 mM DTT and 10% v/v glycerol) and applied to columns containing 1.25 mL methotrexate-agarose beads (Sigma). The columns were washed with 20 mL of Methotrexate Equilibration Buffer then eluted in 1.5 mL fractions using Methotrexate Elution Buffer (Methotrexate Equilibration Buffer + 4 mM DHF).

We attempted to express and purify *P*. *falciparum* DHFR and pyrimethamine-resistant *P*.*vivax* DHFR under multiple conditions, but were unable to recover stable proteins for enzyme assays.

### Enzyme Assays

DHFR catalyzes the reduction of dihydrofolate (DHF) to tetrahydrofolate (THF), coupled to the oxidation of nicotinamide adenine dinucleotide phosphate (NADPH). This reaction was followed *in vitro* by absorbance at λ_abs_ = 340 nm by spectrophotometry (SpectraMax 190, Molecular Devices) in UV-transparent 96-well plates (Costar, Corning) at 21 °C. NADPH and DHF absorb at 340 nm with a combined extinction coefficient of ε^340nM^ = 12,300 M^−1^ cm^−1^, which takes into consideration the oxidation of NADPH and the reduction of DHF.

*H*. *sapiens* DHFR (*Hs*DHFR) and *P*. *vivax* DHFR (*Pv*DHFR) were thawed and stored at 4 °C for use in the enzyme assay. DHF and NADPH stocks were made to 1 mM in assay buffer (0.1 M Tris, pH 7.4) and stored at −20 °C until use. Triclosan (Irgasan; Sigma-Aldrich) (stored as a 1 mM stock in 10% (v/v) DMSO in 0.1 M Tris, pH 7.4) was incubated with *Hs*DHFR or *Pv*DHFR for 10 minutes in assay buffer (0.1 M Tris, pH 7.4) before addition of 100 μM DHF and 100 μM NADPH. Reaction conditions: [triclosan] = 0 or 100 µM, [*Hs*DHFR] = 30.5 nM, [*Pv*DHFR] = 18.5 nM, [DHF] = [NADPH] = 100 µM.

Initial rates of reaction at various concentrations of triclosan were monitored by spectrophotometry (SpectraMax 190, Molecular Devices) and data were analyzed using Excel (Microsoft). Initial rates were determined by following the linear absorbance decrease and used to derive IC_50_ values. Reaction turnover was assayed in UV-transparent 96-well plates (Costar, Corning) with a total volume of 100 μL. DHF and NADPH stocks were made up to 1 mM in assay buffer (0.1 M Tris, pH 7.4) and stored at −20 °C until use.

To deal with variations in enzyme activity in different preparations, kinetic data were normalized as relative activities and fitted to the following equation:

$$ \% \,{\rm{activity}}= \% \,{{\rm{activity}}}_{{\rm{\max }}}/(1+[{\rm{triclosan}}]/{{\rm{IC}}}_{50}),$$

assuming competitive inhibition and using the Cheng-Prusoff equation^36^. Errors were calculated from multiple kinetic runs. The kinetics of *Pv*DHFR carried a larger error than *Hs*DHFR because *Pv*DHFR was prone to loss of activity during purification and storage. Duplicates were performed and IC_50_ values were obtained by fitting the data shown in Fig. 1C to the above equation (MatLab software, Mathworks – curves were fitted by Fabrice Gielen, Cambridge).

Enzyme molarity calculations were based on the following sequences:

*Hs*DHFR: GSMVGSLNCIVAVSQNMGIGKNGDLPWPPLRNEFRYFQRMTTTSSVEGKQN LVIMGKKTWFSIPEKNRPLKGRINLVLSRELKEPPQGAHFLSRSLDDALKLTE QPELANKVDMVWIVGGSSVYKEAMNHPGHLKLFVTRIMQDFESDTFFPEIDL EKYKLLPEYPGVLSDVQEEKGIKYKFEVYEKND.

*Pv*DHFR:

GSMEDLSDVFDIYAICACCKVAPTSEGTKNEPFSPRTFRGLGNKGTLPWKCNS VDMKYFSSVTTYVDESKYEKLKWKRERYLRMEASQGGGDNTSGGDNTHGG DNADKLQNVVVMGRSSWESIPKQYKPLPNRINVVLSKTLTKEDVKEKVFIIDS IDDLLLLLKKLKYYKCFIIGGAQVYRECLSRNLIKQIYFTRINGAYPCDVFFPEF DESQFRVTSVSEVYNSKGTTLDFLVYSKVGG.

### Culture of wild-type and transgenic *P*. *falciparum*

*P*. *falciparum* strain NF54 was transfected with a pDC10 derivate^37^ and cultivated as described previously^38^. Wild-type and transgenic parasites were cultivated in the presence of increasing concentrations of triclosan (0–2.6 µM) or pyrimethamine (0–35 nM). Drug treatment of cultures was started at 0.5–1% parasitemia (trophozoite stage) and run for 48 h. Parasitemias were quantified using the SYBR method^39^ and resulting fluorescence data were analysed and plotted using Excel. The experiments were done twice in biological triplicates.

### Docking

The starting coordinates of the human DHFR (PDB ID: 3NTZ), *P*. *vivax* DHFR (PDB ID: 2BL9), *P*. *falciparum* DHFR (PDB ID: 4DPD), *P*. *vivax* double mutant DHFR (PDB ID: 2BLA), and *P*. *falciparum* quadruple mutant DHFR (PDB ID: 4DP3) were collected from the Protein Data Bank (www.rcsb.org)^40–42^ and employed in Glide (Small-Molecule Drug Discovery Suite 2014-2: Glide, version 6.3, Schrödinger, LLC, New York, NY, 2014) docking calculations.

For Glide calculations, all the crystallographic structures were first imported to Maestro (Schrödinger Release 2014-2: Maestro, version 9.8, Schrödinger, LLC, New York, NY, 2014). With the exception of NADPH, all co-crystallized ligands and water molecules were identified and removed from the structures and the enzymes were treated using the Protein Preparation Wizard (Schrödinger Release 2014-2: Schrödinger Suite 2014-2 Protein Preparation Wizard; Epik version 2.8, Schrödinger, LLC, New York, NY, 2014) provided by Maestro. All the enzymatic structures were also checked for missing atoms, bonds, and contacts. The structure of triclosan was first constructed using Maestro’s building tool and energetically minimized for processing using LigPrep, which allowed the acquisition of a valid low-energy 3D structure for this inhibitor with correct protonation state in the pH range of 7 ± 2. Molecular Docking was initiated by generating a Grid file using Receptor Grid Generation tool of Glide. Grid files containing receptor and binding site information required for molecular docking were prepared using the default options of the Receptor Grid Generation tool with the grid box being centered at the coordinates average of the co-crystallized ligand for each crystallographic structure. To test the reliability of the docking procedure, all the co-crystallized ligands were first docked into the corresponding crystallographic structure using the Extra Precision (XP) Glide algorithm. Subsequently, triclosan was also successfully docked in each active site and the lowest energy conformation provided by Glide Score was chosen.

### Ethics statement

Human blood and plasma were obtained from the local blood bank and ethical clearance for using this blood for this research was granted by the Ethics Committee of the Institute of Biomedical Sciences at the University of São Paulo (No. 842/2016).
